# Effects of transcranial direct current stimulation over human motor cortex on cognitive-motor and sensory-motor functions

**DOI:** 10.1038/s41598-023-48070-z

**Published:** 2023-11-28

**Authors:** Aoun Rizvi, Kara Bell, Daniel Yang, Maria P. Montenegro, Hakjoo Kim, Shancheng Bao, David L. Wright, John J. Buchanan, Yuming Lei

**Affiliations:** Program of Motor Neuroscience, Department of Kinesiology and Sport Management, Texas A&M University, College Station, TX 77843 USA

**Keywords:** Human behaviour, Motor control

## Abstract

The primary motor cortex (M1) is broadly acknowledged for its crucial role in executing voluntary movements. Yet, its contributions to cognitive and sensory functions remain largely unexplored. Transcranial direct current stimulation (tDCS) is a noninvasive neurostimulation method that can modify brain activity, thereby enabling the establishment of a causal link between M1 activity and behavior. This study aimed to investigate the online effects of tDCS over M1 on cognitive-motor and sensory-motor functions. Sixty-four healthy participants underwent either anodal or sham tDCS while concurrently performing a set of standardized robotic tasks. These tasks provided sensitive and objective assessments of brain functions, including action selection, inhibitory control, cognitive control of visuomotor skills, proprioceptive sense, and bimanual coordination. Our results revealed that anodal tDCS applied to M1 enhances decision-making capacity in selecting appropriate motor actions and avoiding distractors compared to sham stimulation, suggesting improved action selection and inhibitory control capabilities. Furthermore, anodal tDCS reduces the movement time required to accomplish bimanual movements, suggesting enhanced bimanual performance. However, we found no impact of anodal tDCS on cognitive control of visuomotor skills and proprioceptive sense. This study suggests that augmenting M1 activity via anodal tDCS influences cognitive-motor and sensory-motor functions in a task-dependent manner.

## Introduction

The primary motor cortex (M1) is widely recognized as a somatotopic assembly of upper motor neurons, which have substantial disynaptic and monosynaptic connections to lower motor neurons in the spinal cord^1,2^. These spinal motoneurons, in turn, precisely regulate muscle activity, enabling a wide variety of skilled movements^3^. Given this structural architecture, M1 is predominantly acknowledged for its primary role in the execution of voluntary movements. Additionally, M1 has dense anatomical interconnections with other brain regions, particularly with the primary somatosensory area (S1)^4^, the premotor area (PMA)^5^, and the supplementary motor area (SMA)^6^. S1 is essential in processing incoming sensory information^7^, while PMA and SMA are implicated in cognitive processes such as planning, selection, and coordination of motor actions^8–11^. Therefore, motor command signals in M1 are likely inherently intertwined with cognitive and sensory signals such as attention, decision-making, and sensory processing.

Emerging evidence indicates that the function of M1 extends beyond merely commanding muscle activity, it also processes nonmotor signals^12^. Neuroimaging and neurophysiological studies have shown that M1 activity is profoundly affected by the focus of attention^13–15^, perceptual decisions^16,17^, and afferent inputs^18–21^. It is noteworthy that intrinsic fluctuations of M1 activity are, to some extent, associated with ongoing cognitive and sensory processes. Animal studies reveal that changes in M1 population activity correlate with arousal-like cognitive processes^22^. Human studies show that M1 excitability is distinctly modulated by ongoing tactile and proprioceptive inputs^23^. These findings suggest that M1 activity does not simply reflect the output of completed top-down cognitive processing and/or bottom-up feedback processing^24^. Rather, it reflects a dynamic interplay between ongoing motor and nonmotor processes. As such, the manipulation of M1 activity could concurrently influence the processing of cognitive and sensory signals involved in motor behaviors.

Brain stimulation techniques have been extensively employed to decipher the functional significance of specific brain regions and to establish causal connections between these areas and behavior^25–27^. For example, Thura and Cisek^28^ revealed that subthreshold microstimulation of the M1 in monkeys, timed just before a choice was made, led to an elongated decision-making process, implying a direct causal role of M1 in action selection. Derosiere^29^ et al. investigated M1's role in the context of value-based motor decision-making in humans. By utilizing repetitive transcranial magnetic stimulation (rTMS), they found that stimulation of the left M1 modified the implementation of an underlying value-based rule during decision-making tasks, while stimulation of the right M1 improved adherence to this rule. Transcranial direct current stimulation (tDCS) distinguishes itself among brain stimulation techniques as a non-invasive method that modulates cortical excitability through the administration of weak direct currents^30,31^. Notably, tDCS is often imperceptible to subjects, particularly when lower intensities below 1.5 mA are used in combination with large electrodes and saline solutions to minimize charge density^32–34^. This makes it challenging for subjects to differentiate between actual and sham stimulation, thus preserving the integrity of blinding in experimental designs^32–34^. The ease with which tDCS electrodes can be secured to the scalp also provides subjects with the freedom to move their heads and bodies during stimulation^34^, adding to its versatility in various experimental setups.

Depending on the polarity of the applied stimulation, tDCS can either enhance or inhibit cortical excitability. Specifically, anodal tDCS tends to increase cortical excitability, while cathodal tDCS typically decreases it. Human studies have revealed that anodal tDCS over M1 increases the amplitude of motor evoked potentials (MEPs)^35^, indicating a heightened corticospinal excitability. Such excitability is associated with the alpha rhythm observed over M1 and the magnitude of the BOLD activation response^36,37^. In contrast, cathodal tDCS over M1 leads to a reduction in MEP amplitude^35^. Animal studies have similarly shown that tDCS induces polarity-specific changes in M1 excitability, with anodal tDCS increasing and cathodal tDCS decreasing MEP amplitude^38^. Anodal tDCS is thought to produce effects that resemble long-term potentiation (LTP), whereas cathodal tDCS is considered to produce effects analogous to long-term depression (LTD). The effect of tDCS on LTP and LTD mechanisms is associated with the alteration of voltage-dependent ion channels^39^ and the activity of NMDA receptors^40,41^. The application of tDCS has centered on its neuromodulatory after-effects following either anodal or cathodal stimulation protocols^42^. By examining behavioral changes following tDCS, researchers can assess the subsequent impacts of tDCS on behavior. However, offline tDCS gives the brain's neural networks sufficient time to adapt to the stimulation, which might mask the real-time effects of tDCS-induced changes in neural excitability on task performance. Conversely, online tDCS protocols are designed to modify neural excitability in ways that are specifically relevant to the task, context, and timing. This method is more effective in exploring the direct causal relationship between changes in neural excitability and behavioral outcomes. In our study, we applied anodal tDCS to M1 while participants simultaneously engaged in standardized motor tasks that required cognitive and sensory processing. Our objective was to examine the impact of modulating M1 excitability in real-time on cognitive-motor and sensory-motor functions within the context of these tasks.

This study utilized a series of established, standardized robotic tasks that allow sensitive and objective measurements of various cognitive-motor and motor-sensory functions. Specifically, in the "Object Hit & Avoid" task, participants were instructed to hit two designated targets while avoiding all other objects within their workspace^43–45^. This task served as an evaluation of participants' action selection and motor inhibitory control. In the "Ball on Bar" task, participants moved a virtual ball placed on a bar that linked both hands^45–47^. The task required participants to synchronize their hand movements to guide the ball to hit designated targets. This task served as an assessment of the participants' bimanual coordination skills. In the "Reverse Visually Guided Reaching" task, participants were required to exert sustained cognitive control to direct movements in the opposite direction of the target's location^48–50^. This task served to evaluate the participants' ability to control visuomotor skills based on predefined rules. In the "Arm Position Matching" task, one of the participant's arms was passively and randomly positioned, and they were then asked to actively mirror this position using their other arm^51–53^. This task served to assess the participants' somatosensory processing skills, particularly in relation to limb position sense. Drawing on the extensive research detailed earlier, which highlights the complex role of M1 in integrating motor and non-motor functions, we hypothesize that concurrently augmenting M1 excitability with anodal tDCS while participants engaged in these robotic tasks will lead to enhancements in performance on tasks that require intricate cognitive-motor and motor-sensory integrations.

## Results

### Impact of M1 stimulation on action selection and motor inhibitory control

**Figure 1:**
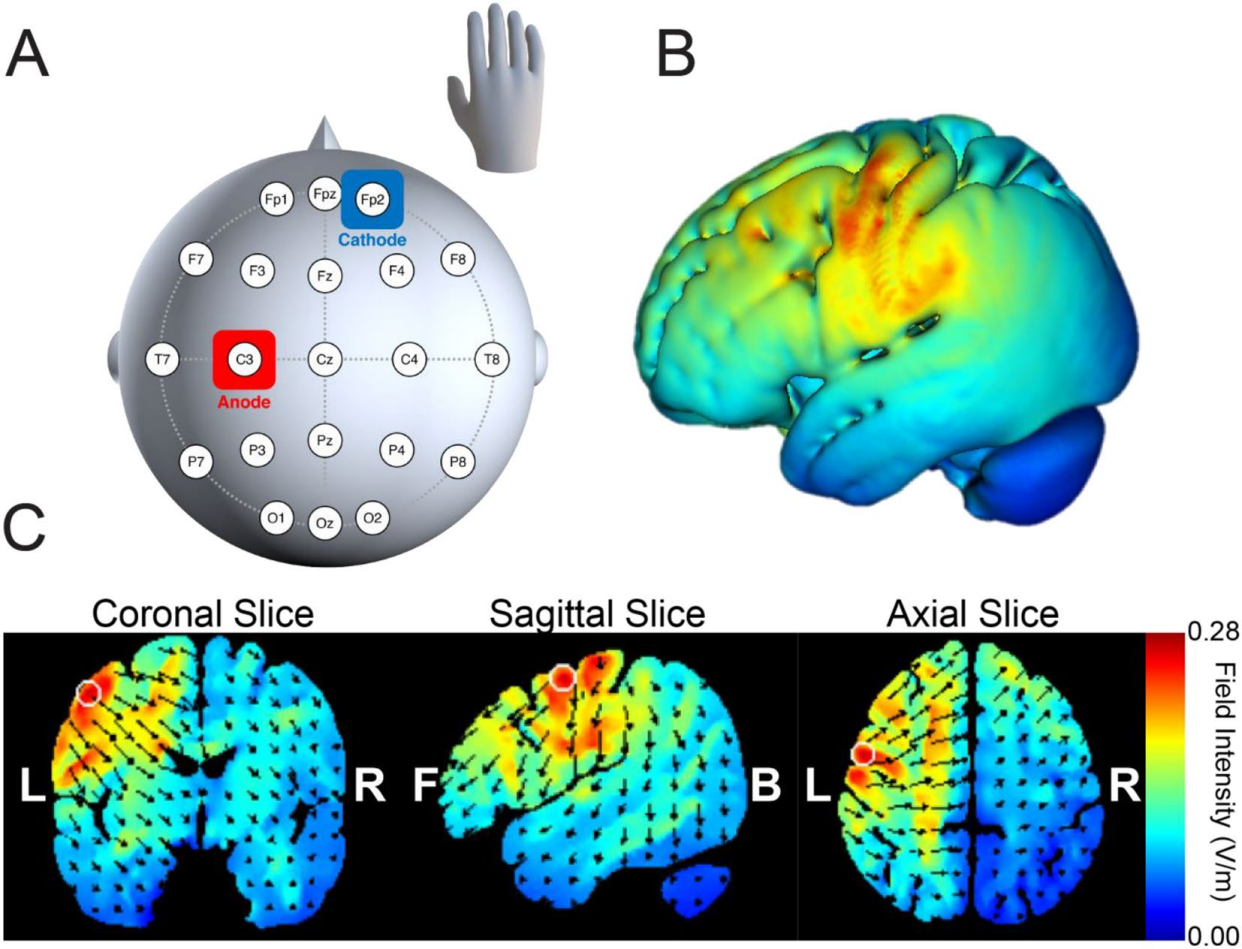
tDCS electrode placement during robotic tasks. (**A**) In the anodal-tDCS group, the anode was placed over the left M1, precisely aligned with the C3 location based on the International 10/20 System. The cathode was positioned on the right supraorbital area. In the sham-tDCS group, the electrode setup remained unchanged; however, stimulation lasted only for 30 s at the beginning and end of the robotic tasks. (**B, C**) The current flow associated with this electrode arrangement was simulated using HD-ExploreTM (Soterix Medical Inc., New York, NY). A heightened current distribution was noted in the area corresponding to the left M1.

**Figure 2:**
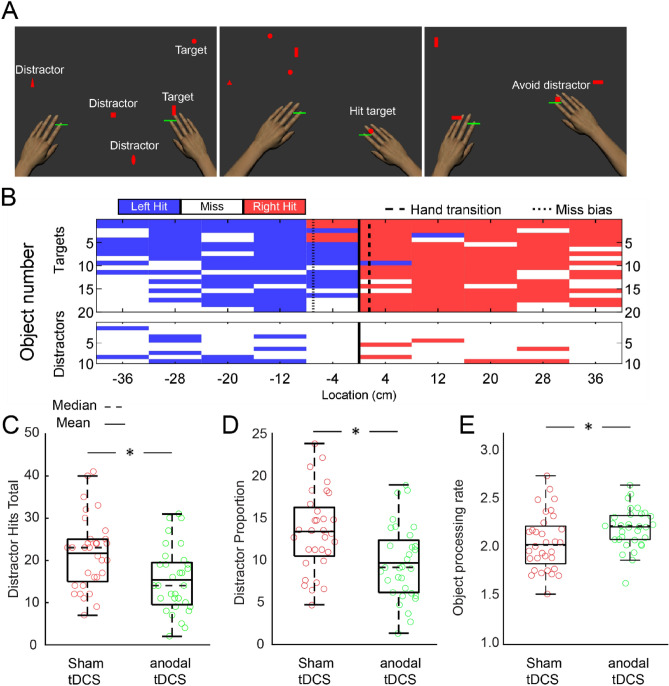
Participant performance and boxplots for the "Object Hit & Avoid" task. (**A**) Display of a participant actively engaged in the "Object Hit & Avoid" task. The presented objects comprise two targets and six distractors. (**B**) Task performance summary for a representative participant. The y-axes represent the number of targets (top), or distractors (bottom) dropped from each bin, as displayed on the x-axis. Hits executed with the left hand are visualized in blue, while hits with the right hand are in red. Missed objects appear in white. The beginning of the task is denoted by the top of each graph, while the end is represented by the bottom. Hand transitions are marked with dashed lines, and missed biases are highlighted with dotted lines. (**C–E**) The boxplots illustrate Distractor Hits (**C**), Distractor Proportion (**D**), and Object Processing Rate (**E**). Individual data points are represented by open circles, with the anodal-tDCS group in green and the sham-tDCS group in red.

Numerous studies have established that the standardized "Object Hit & Avoid" robotic task is an effective method for evaluating cognitive-motor functions such as action selection and motor inhibitory control^43–45^. In this task, anodal tDCS was administered to the left M1 (Fig. 1), with participants being instructed to hit two designated targets while avoiding all other distractor objects within their workspace (Fig. 2A). Figure 2B presents the distribution of hits and misses for target and distractor objects for a representative participant. The participant demonstrated a high level of accuracy in hitting targets and successfully avoiding distractor objects. The anodal-tDCS group exhibited a reduction in distractor hits (Distractor Hit = 15.4 ± 7.7; t(62) = 3.08; p = 0.002; Cohen's d = 0.77; Fig. 2C), indicating a decrease in the total number of distractor objects contacted, and displayed a diminished proportion of distractor hits (Distractor Proportion = 9.7 ± 4.4; t(62) = 3.24; p < 0.001; Cohen's d = 0.81; Fig. 2D), representing a lower ratio of distractors hit to total objects hit, in contrast to the sham-tDCS group (Distractor Hit = 21.6 ± 8.6; Distractor Proportion = 13.4 ± 4.8). The object processing rate, a measure of the rate at which objects are accurately processed (calculated as the sum of the number of targets hit and distractors missed per second), was significantly higher in the anodal-tDCS group compared to the sham-tDCS group (t(62) = 2.17; p = 0.017; Cohen's d = 0.54; Fig. 2E). Specifically, the anodal-tDCS group had a processing rate of 2.20 ± 0.21 objects/second, whereas participants in the sham-tDCS group processed at a rate of 2.04 ± 0.30 objects/second.

### Impact of M1 stimulation on bimanual coordination

**Figure 3:**
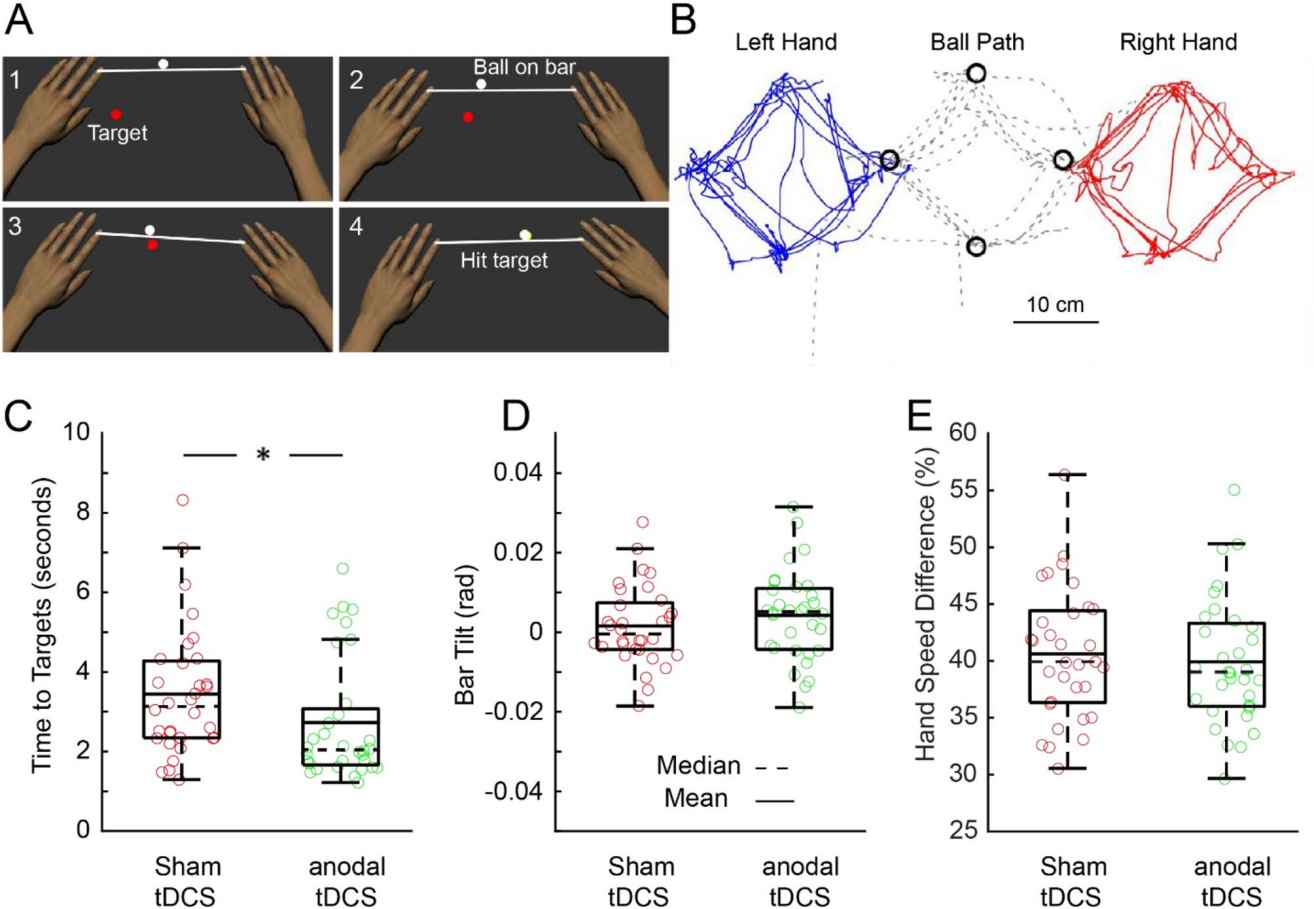
Participant performance and boxplots for the "Ball on Bar" task. (**A**) Display of a participant actively engaged in the "Ball on Bar" task. (**B**) Depiction of hand and ball paths from a representative participant. Solid lines represent the hand paths of the left (blue) and right (red) hands, while the dotted trace indicates the ball's path. (**C–E**) The boxplots illustrate Time to Targets (**C**), Bar Tilt (**D**), and Hand Speed Difference (**E**). Individual data points are represented by open circles, with the anodal-tDCS group in green and the sham-tDCS group in red.

The "Ball on Bar" robotic task is a commonly employed method for evaluating performance on a bimanual activity^45–47^. The main objective of the task is to hit as many targets as possible through coordinated movements of both limbs (Fig. 3A). The task is designed to have three escalating levels of difficulty. At the foundational level, the ball is fixed at the bar's midpoint. In the intermediate level, the ball's position becomes dependent on the bar's inclination; it stays central when the bar is level but shifts towards the edge as the tilt increases, dropping off when the angle surpasses 20 degrees. In the most challenging level, the ball is unrestrained and permitted to roll freely along the bar. Our investigation focuses on this ultimate level due to its closer resemblance to the real-life dynamics of ball movement on a surface. Figure 3B depicts the trajectories of the ball and hand for a representative participant at the most challenging level of the task. Notably, the participant reached each target with corrective movements. The patterns of hand movements closely mirrored the movements of the ball. The "Time to Target", defined as the total time elapsed from when targets appeared to when the ball reached the targets, was less in the anodal-tDCS group (Time to Target = 2.7 ± 1.5 s; t(62) = 1.97; p = 0.027; Cohen's d = 0.49; Fig. 3C) compared to the sham-tDCS group (Time to Target = 3.4 ± 1.6 s). However, there was no significant difference in "Bar Tilt", determined by the absolute angle of the bar relative to the frontal plane, and "Hand Speed Difference", which represents the cumulative sum of the absolute difference in speed between the two hands, between the anodal-tDCS group and the sham-tDCS group (Bar Tilt: p = 0.2, Fig. 3D; Hand Speed Difference: p = 0.3, Fig. 3E).

### Impact of M1 stimulation on cognitive control of visuomotor skills

**Figure 4:**
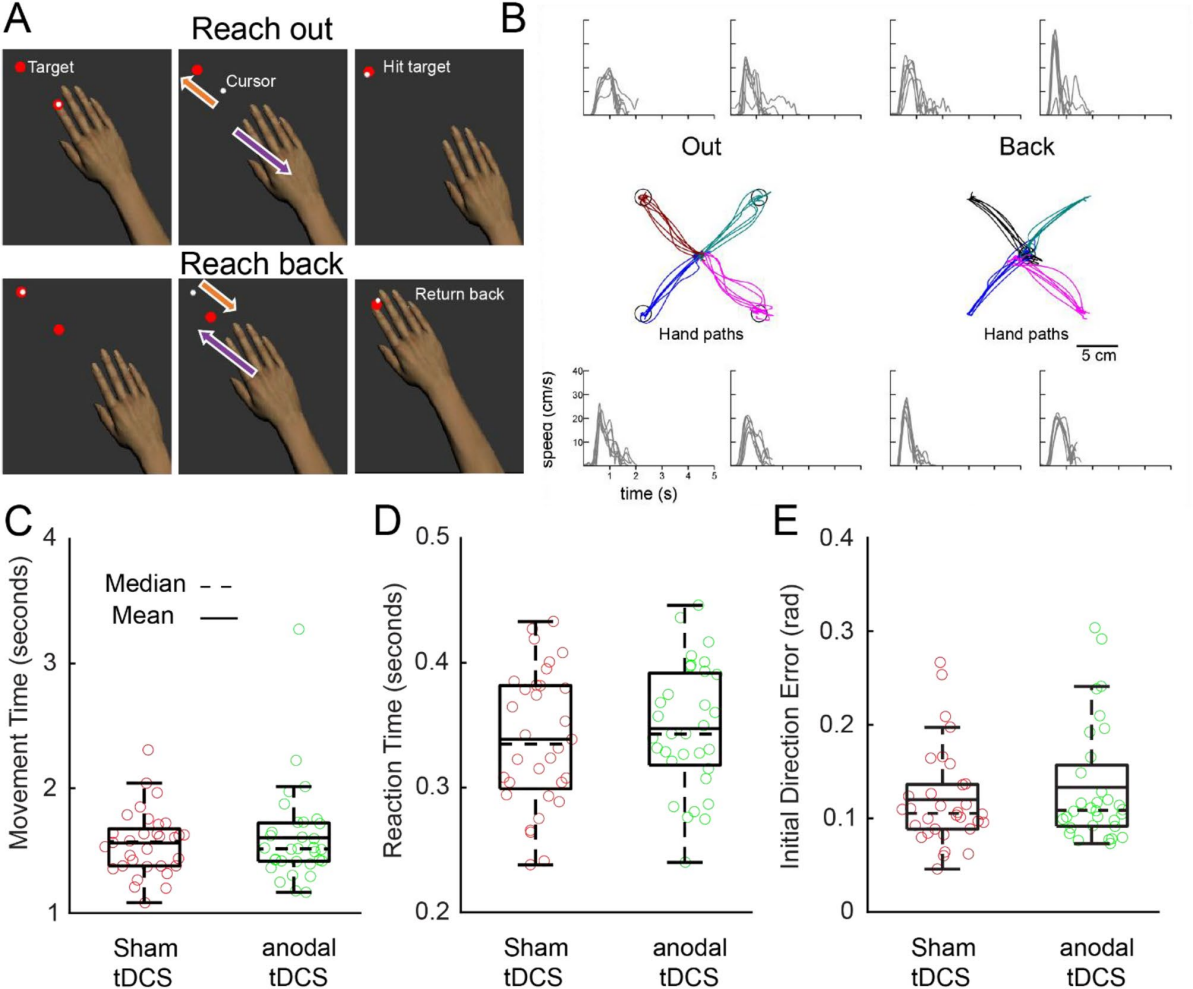
Participant performance and boxplots for the "Reverse Visually Guided Reaching" task. (**A**) The top images display the reach-out phase, while the bottom ones represent the reach-back phase. It's noteworthy that the white feedback dot for the hand moves in the opposite direction to the actual hand movement. (**B**) A depiction of hand trajectories and hand speed profiles in a representative participant. (**C–E**) The boxplots illustrate Movement Time (**C**), Reaction Time (**D**), and Initial Direction Error (**E**). Individual data points are represented by open circles, with the anodal-tDCS group in green and the sham-tDCS group in red.

The "Reverse Visually Guided Reaching" robotic task is widely used for studying cognitive control of motor actions^48–50^. This task demands cognitive control to regulate arm movement in the opposite direction of the target (Fig. 4A). Additionally, reverse reaching demands ongoing cognitive control to assist real-time control of limb movements, thereby directing the cursor towards the target. Prior studies indicate that individuals with cognitive impairments typically exhibit greater variability in their reach trajectories during the task, and often experience less success in accurately reaching the intended targets. Figure 4B depicts the hand paths when reaching out and back from the targets, along with the speed profiles for multiple reaches to each target in a representative participant. No significant differences were found in parameters such as "Movement Time", denoting the entire duration of the movement, and "Reaction Time", the interval between the target's appearance and the commencement of the movement, between the anodal-tDCS group and the sham-tDCS group (Movement Time: p = 0.3, Fig. 4C; Reaction Time: p = 0.3, Fig. 4D). Similarly, there was no difference in "Initial Direction Error," which quantifies the angular deviation, between the two groups (p = 0.2, Fig. 4E).

### Impact of M1 stimulation on limb position sense

**Figure 5:**
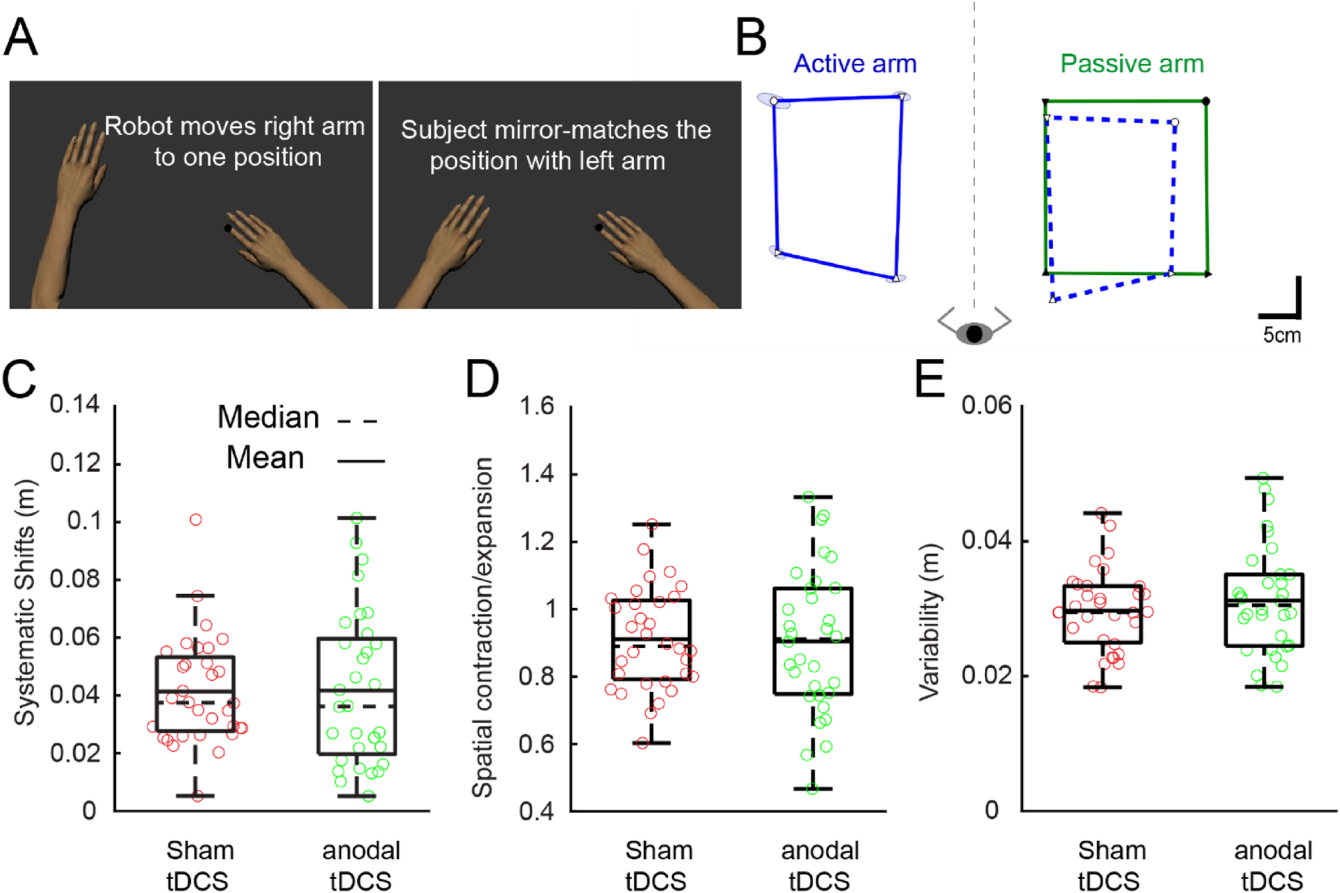
Participant performance and boxplots for the "Arm Position Matching" task. (**A**) The robot actively directs the right arm to four distinct spatial locations while the participant mirrors the movement using the left arm. (**B**) Data from the right arm is mirror transformed to assess differences between the position of the hand moved passively by the robot and the actively moved hand. The ellipses around the target points represent the standard deviation. (**C–E**) The boxplots illustrate Systematic Shifts (C), Spatial Contraction/Expansion (**D**), and Variability (**E**). Individual data points are represented by open circles, with the anodal-tDCS group in green and the sham-tDCS group in red.

The "Arm Position Matching" robotic task has been established as a reliable, quantitative assessment method for multi-joint limb position sense^51–53^. During this task, the participant's right arm is passively and randomly positioned, after which they are asked to actively mirror this position using their left arm (Fig. 5A). Figure 5B depicts an example of matching behavior by a representative participant. Overall, the participant's active arm (blue lines) closely mirrors the position of the passive arm (green lines). This participant exhibited low variability across trials and showed minimal spatial contraction/expansion or systematic shifts. No significant differences were observed between the anodal-tDCS and sham-tDCS groups in terms of "Systematic Shifts" (representing consistent differences between the positions of the active and passive arms) and "Spatial Contraction/Expansion" (measured by calculating the area spanned by the active hand for the targets and normalizing this by the total spatial area covered by the passive hand) (Systematic Shifts: p = 0.5, Fig. 5C; Spatial Contraction/Expansion: p = 0.4, Fig. 5D). Likewise, "Variability" (calculated as the standard deviation of the active hand's position for each target location) showed no significant difference between the two groups (p = 0.2, Fig. 5E).

## Discussion

Our study examined the online effects of anodal-tDCS applied over M1 on cognitive-motor and sensory-motor functions. We employed four specially designed robotic tasks to evaluate various aspects of these functions, including action selection, motor inhibitory control, bimanual coordination, cognitive control of visuomotor skills, and the sense of limb position. Our findings reveal that anodal tDCS over M1 distinctly affects behavioral performance across these tasks. Specifically, the application of anodal tDCS over M1 results in enhanced motor inhibitory control and action selection capabilities. Furthermore, it leads to a decrease in the time required to execute bimanual movements, which implies improved bimanual coordination. In contrast, anodal tDCS over M1 does not impact cognitive control of visuomotor skills or limb position sense, indicating that these cognitive and sensory-motor functions remain unaffected. Therefore, our research suggests that anodal tDCS-driven enhancement of M1 activity influences cognitive-motor and sensory-motor functions in a task-dependent manner.

The ability to select appropriate motor actions while concurrently inhibiting inappropriate ones is fundamental to human behavior. M1 plays an essential role in motor action selection and inhibitory control^54,55^. Previous studies have indicated that selecting between various motor actions can significantly alter neural activity in motor cortical areas, including M1^56,57^. Neural signals from cortical regions such as SMA, PMA, and M1 can activate inhibitory interneurons in the spinal cord, suggesting a potentially significant role for M1 in motor activity inhibition^58^. Damage to M1 can lead to spasticity, a condition characterized by the loss of motor inhibition^59,60^. Furthermore, recent evidence suggests that the neuromodulation of M1 plasticity through ultrasound stimulation can influence inhibitory control function^61^. In this study, we aimed to examine the online modulatory effects of applying tDCS to M1 on action selection and inhibitory control. We used an "Object Hit & Avoid" robotic task that required participants to hit specified targets while avoiding all other distractor objects in their environment. We quantitatively measured participants' ability to rapidly select appropriate motor actions using the object processing rate, which calculated the speed at which objects were accurately processed. We found that participants in the anodal-tDCS group displayed a significantly faster processing rate compared to the sham-tDCS group. Also, the anodal-tDCS group showed fewer distractor hits and a lower proportion of distractor hits compared to the sham-tDCS group. These findings suggest that applying anodal-tDCS to M1 enhances action selection and inhibitory control capabilities.

Neurophysiological studies have indicated that bimanual movement involves neuronal activity not only in the dorsal premotor cortex and SMA^62–66^, but also in M1^65,67^. The SMA is believed to be more involved in the coordination between arms, and its activity is modulated based on the complexity of the coordination^68^. In contrast, M1 activity during bimanual movements is more closely linked to motor execution. This difference is supported by primate studies which indicate a greater shift in neural representations in the SMA between unimanual and bimanual motor actions^63,64^, while M1's representation remains relatively consistent between unimanual and bimanual motor actions^69^. To explore the impact of anodal-tDCS over M1 on bimanual movement, we employed a "Ball on Bar" robotic task. This task required participants to hit targets through coordinated movements of both limbs. We found that participants in the anodal-tDCS group exhibited better performance in bimanual execution metrics, such as faster movement time. However, there was no significant difference in bimanual coordination metrics, such as the absolute tilt of the bar and the absolute difference in speed between the two hands. These findings suggest that the application of anodal-tDCS over M1 enhances the execution of bimanual movements, but not the coordination of movements between the arms.

While we observed that anodal-tDCS applied to the M1 improved performance in the "Object Hit & Avoid" and "Ball on Bar" tasks, we did not find the same enhancement in the "Reverse Visually Guided Reaching" and "Arm Position Matching" tasks. The " Reverse Visually Guided Reaching" task is a complex motor task that requires the generation of a cognitive rule to initiate and control hand movement in the opposite direction of the target. It also demands ongoing cognitive control to assist in real-time hand movement. Successful completion of this task necessitates the involvement of a highly distributed brain network, including fronto-parietal circuits, the basal ganglia, the cerebellum, and M1^48,70,71^. Impairments in this task have been observed across various neurological injuries, including stroke^48^, Alzheimer’s Disease^72^, multiple sclerosis^73^, epilepsy^45^, amyotrophic lateral sclerosis^74^, and concussion^75^. This suggests that any impairment within these neural networks could disrupt the complex cognitive and motor processes necessary to complete the "Reverse Visually Guided Reaching" task. As this task necessitates rule-based cognitive control, augmenting M1 activity through anodal tDCS may not translate into marked improvements in task execution. The "Arm Position Matching" task was designed to evaluate participants' somatosensory processing capabilities, with a particular emphasis on the sense of limb position. The processing of somatosensory signals requires the coordination of multiple neural pathways, including the thalamus, S1, and M1. The thalamus serves as a critical relay center, receiving and processing somatosensory information from peripheral receptors and then projecting this information to the relevant areas of the cortex^76^. S1 is the first stage of somatosensory processing in the cortex^77^, which processes the magnitude and quality of sensory inputs, such as skin pressure and vibration, joint angle and speed, and texture, and relays this information to M1. M1 receives sensory inputs via polysynaptic thalamocortical connections and monosynaptic S1-M1 connections^78,79^. M1 interacts with the thalamus and S1 to integrate somatosensory feedback for movement control. Therefore, the absence of significant performance enhancement in the "Arm Position Matching" task in the anodal tDCS group could be attributed to the fact that M1's primary role isn't in the basic processing of sensory signals. Instead, M1 receives processed sensory signals for the purpose of sensorimotor control.

In this study, we leveraged standardized robotic tasks to explore the online effects of anodal tDCS on cognitive-motor and sensory-motor functions. The use of these robotic tasks allows for objective and sensitive measurements of behavioral performance that can detect subtle changes induced by brain stimulation. Additionally, these tasks produce continuous variables, thus avoiding floor or ceiling effects, and have demonstrated excellent test–retest reliability, establishing themselves as reliable tools for assessing cognitive-motor and sensory-motor functions^43–53^. However, our study's design, which involves movements constrained to the horizontal plane and obscures participants' arms from view, introduces additional layers of complexity. Although it provides an immersive environment, this setup may compound the challenges associated with cognitive, sensory, and motor interactions. Another limitation of our study was the reliance on the 10–20 EEG system for the localization of M1 rather than utilizing TMS for more accurate M1 positioning. This method might not have adequately considered the anatomical differences between individuals^80^. Moreover, the lack of neurophysiological measures like motor evoked potential (MEP) in our study precluded the confirmation of whether anodal tDCS elicited the expected increase in M1 excitability. It is also essential to recognize the significance of individual variability in response to tDCS and the variations in electric fields induced by tDCS^81^. In summary, our study reveals significant task-dependent variations in cognitive-motor and sensory-motor performance, elicited by anodal tDCS. Yet, more in-depth research is required to understand how these immediate effects translate into long-term offline effects. Our findings have potential implications for rehabilitation efforts aimed at restoring cognitive, sensory, and motor functions.

## Materials and methods

### Participants

Sixty-four healthy individuals, all right-handed (Age = 20.9 ± 1.8; 38 females and 26 males), with no history of neurological or musculoskeletal disorders, were enrolled in this study. All participants were naïve to our apparatus, the paradigm, and the purpose of the study. All experimental protocols were approved by the Institutional Review Board of Texas A&M University. All subjects gave written informed consent prior to participation, which was approved by the local ethics committee at Texas A&M University in accordance with the Declaration of Helsinki.

### Apparatus

We utilized a bilateral robotic exoskeleton, KINARM (BKIN Technologies Ltd, Kingston, ON, Canada), for performing the robotic tasks and collecting movement data in this study. The participants were seated on a height-adjustable chair, with their arms horizontally supported by the KINARM exoskeleton. The exoskeleton's linkages were adjusted to custom-fit each participant based on their arm length and geometry. The KINARM was integrated with a virtual reality system that projected visual targets on a horizontal display, aligning them on the same plane as the arms. The chair was then positioned such that the arms were under the horizontal display. To exclude any direct visual feedback, the view of the arms was obstructed, and a white cursor was displayed, signifying the location of the index fingertip, to guide the participant's movement. The 2-D position of the index fingertip was sampled at a frequency of 1000 Hz, low-pass filtered at 15 Hz.

### Robotic tasks

All robotic tasks were performed on the KINARM exoskeleton. The study incorporated four standardized tasks: the Object Hit & Avoid task, which evaluated participants' action selection and motor inhibitory control; the Ball on Bar task, which was designed to test cooperative bimanual coordination; the Reverse Visually Guided Reaching task, which aimed to measure cognitive control of visuomotor skills; and the Arm Position Matching task, which assessed limb position sense without the aid of visual feedback. These tasks were specifically chosen to collectively provide a comprehensive evaluation of different cognitive-motor and sensory-motor functions.

#### Object hit and avoid task

As the task began, participants were shown two shapes on the screen. They were instructed to hit these shapes, defined as 'targets', while avoiding contact with all other shapes, defined as 'distractors'. Participants controlled two horizontal paddles, representing their hands, to hit the targets and avoid the distractors. The task consisted of 10 virtual bins from which both target and distractor objects were dropped. Each bin released a total of 30 objects, comprising 20 targets and 10 distractors (200 targets and 100 distractors in total). As the task progressed, the maximum number of objects that could appear concurrently on the screen increased incrementally from 1 to 16. Furthermore, the dropping speed of the objects increased over the course of the task, starting at approximately 10 cm/s, and reaching roughly 50 cm/s by the end.

#### Ball on bar task

Participants were required to control a virtual ball that was placed on a bar linking both hands. The task required participants to synchronize their hand movements to guide the ball to reach designated targets. The targets initially appeared red, signaling for the participant to 'go'. Once the ball was successfully moved inside the target, it turned yellow. Participants had to maintain the ball inside the target for a duration of 1 s, after which the next red target would appear. The difficulty level of the task increased as it progressed. Initially, the ball was fixed at the center of the bar. However, at the most challenging level, the ball could roll freely with no friction on the bar. This required participants to exert more effort to keep the ball on the bar and guide it to the target. This study focused solely on this most difficult level.

#### Reverse visually guided reaching task

Participants used their right hand to initiate a reach towards a central target, represented by a cursor that was synchronized with the movement of their index finger. Upon reaching the central target, the relationship between the cursor and hand movement was inverted, causing the cursor to move 180° opposite to the finger's motion. Following a random time interval ranging from 750 to 1250 ms, one of four peripheral targets appeared. These peripheral targets were spaced 90° apart and located 10 cm from the center. Participants were asked to reach the peripheral target within a 6-s window. Once the cursor reached the target, the central target reappeared, and participants were instructed to guide the cursor back to the center. Targets were presented in six blocks of four trials, with each of the 4 targets appearing randomly within each block.

#### Arm position matching task

Participants were instructed to relax their right arm, referred to as the 'passive hand'. The robot then moved the right hand to one of four different spatial locations following a linear path and a bell-shaped speed profile (maximum speed less than 1 m/s). Once the robotic movement stopped, participants were asked to move their left hand, referred to as the 'active hand', to the mirror location in space, matching the position of their right hand. Participants informed the examiner once they completed each trial, triggering the start of the next trial. The location of the targets was randomized within each block. Participants completed 6 blocks, leading to a total of 24 trials.

### Transcranial direct current stimulation (tDCS)

Participants were allocated into two groups: the anodal-tDCS group (n = 32; Age = 21.0 ± 1.8; 20 females and 12 males) and the sham-tDCS group (n = 32; Age = 20.9 ± 1.8; 18 females and 14 males). The anodal-tDCS group received anodal stimulation that targeted the left M1, whereas the sham group experienced a procedure without active stimulation. The anodal stimulation was administered through a battery-operated device (tDCS Stimulator; TCT Research Limited, Hong Kong), which supplied a current of 1 mA over a duration of 20 min. This current was delivered through a pair of 5 × 5 cm^2^ electrodes, which were covered in saline-soaked sponges, resulting in a current density of 0.04 mA/cm^2^. The anode was positioned over the left M1, precisely aligned over the C3 according to the International 10/20 System, while the cathode was situated on the right supraorbital region (Fig. 1A). This setup is acknowledged for its precision in M1 stimulation, as visualized in the current flow simulation (Fig. 1B). The current flow simulation was generated utilizing HD-Explore™ (Soterix Medical Inc., NY), indicating a heightened current flow within regions identified as the left M1 (Fig. 1C). Regarding the sham-tDCS stimulation, the identical electrode setup was maintained, but the actual stimulation was only delivered for a duration of 30 s at the beginning and end of the robotic tasks. Participants were blind to whether they received anodal or sham stimulation, and the data-collecting experimenter was also unaware of the specific conditions assigned to each participant, thereby maintaining the integrity of the study's blinding.

### Experimental procedure

After obtaining informed consent from all participants, they were randomly assigned to either the anodal-tDCS group or the sham-tDCS group. Once the tDCS setup was completed, the exoskeleton was adjusted to each participant's individual limb lengths, and the system was calibrated to ensure accurate measurements and optimal performance. All participants were given the opportunity to familiarize themselves with the experimental setup and equipment. They practiced reaching movements while seated in the exoskeleton to ensure they felt comfortable with the procedure and equipment. To minimize potential order effects, the order in which the four robotic tasks were presented differed among participants, with the task sequence being pseudo-randomized for each participant. During the execution of these tasks, tDCS (either anodal or sham) was concurrently administered to allow for its direct influence on task performance. The total duration of the four robotic tasks was roughly 20 min, which also determined the duration of the anodal or sham stimulation. A 1-min break was included between each task to provide a short rest for participants and avoid any performance impact due to fatigue. Upon the completion of the training, the tDCS electrodes were promptly removed from each participant's head.

### Data analysis

Three kinematic parameters were calculated for each robotic task. The selection of these parameters was based on their relevance and interpretability within the context of cognitive-motor and sensory-motor performance, ensuring their applicability to the tasks. In the Object Hit & Avoid task, the calculated parameters included: (1) Distractors Hit, which was the total number of distractor objects hit; (2) Distractor Proportion, calculated as the number of distractors hit divided by the total objects hit; and (3) Object Processing Rate (objects/second), which measured the rate of successfully processed objects, calculated by summing the number of targets hit and distractors avoided per second. For the Ball on Bar task, the determined parameters were as follows: (1) Time to Targets, which referred to the total time taken from the moment the target appeared until the ball reached the target; (2) Bar Tilt, a measurement indicating the angle of the bar in relation to the frontal plane; and (3) Hand Speed Difference, which represented the accumulated sum of the absolute difference in the speed of both hands. For the Reverse Visually Guided Reaching task, the parameters calculated were as follows: (1) Movement Time, which referred to the total duration of the entire movement from the onset to the offset, (2) Reaction Time, which was measured as the time interval between the target's appearance and the onset of the movement, and (3) Initial Direction Angle, a parameter describing the angular deviation between a straight line from the hand cursor's position at the onset of the movement to the end of the initial movement, and another straight line from the cursor's position at movement onset to the final target. In the Arm Position Matching task, the calculated parameters comprised of (1) Systematic Shifts, which represented consistent discrepancies between the active and passive hands, (2) Spatial contraction/expansion, determined by calculating the area covered by the active hand for the targets and then normalizing it by the total spatial area spanned by the passive hand, and (3) Variability, which was assessed as the standard deviation of the active hand's position for each target location. All statistical analyses were conducted using SPSS software. Independent t-tests were conducted to determine any significant differences in the kinematic parameters between the anodal-tDCS and sham-tDCS groups. An alpha level of 5% was employed for all statistical analyses.

## Data Availability

The datasets used and/or analyzed during the current study available from the corresponding author on reasonable request.
